# Late assembly of the *Vibrio cholerae* cell division machinery postpones septation to the last 10% of the cell cycle

**DOI:** 10.1038/srep44505

**Published:** 2017-03-16

**Authors:** Elisa Galli, Evelyne Paly, François-Xavier Barre

**Affiliations:** 1 Institute for Integrative Biology of the Cell (I2BC), Université Paris-Saclay, CEA, CNRS, Université Paris Sud, France

## Abstract

Bacterial cell division is a highly regulated process, which involves the formation of a complex apparatus, the divisome, by over a dozen proteins. In the few model bacteria in which the division process was detailed, divisome assembly occurs in two distinct steps: a few proteins, including the FtsZ tubulin-like protein, form a membrane associated contractile ring, the Z-ring, at ~30% of the cell cycle. The Z-ring serves as a scaffold for the recruitment of a second series of proteins, including integral membrane and periplasmic cell wall remodelling enzymes, at ~50% of the cell cycle. Actual septation occupies most of the remaining half of the cell cycle. In contrast, we present evidence suggesting that early pre-divisional Z-rings form between 40 and 50% of the cell cycle and mature into fully assembled divisome at about 80% of the cell cycle in *Vibrio cholerae*. Thus, actual septation is restricted to a very short amount of time. Our results further suggest that late assembly of the divisome probably helps maintain the asymmetric polar organisation of *V. cholerae* cells by limiting the accumulation of a cell pole marker, HubP, at the nascent cell poles.

The propagation of life relies on the ability of cells to multiply by vegetative division. One of the most important rules imposed upon the process is that each daughter cell receives a complete copy of its mother’s genome. In eukaryotes, the necessary coordination between cell division and the replication/segregation cycle of the genetic material is achieved by coupling the assembly and activity of the division apparatus (the divisome) to the formation and activation of the mitotic spindle, the machinery that effects the simultaneous separation of sister chromosomes after replication. In bacteria, which lack a functional equivalent of the mitotic spindle, it is achieved by directing divisome assembly to the low DNA-density zone that develops at mid-cell between chromatid sisters when the concurrent replication and segregation of the chromosomes is sufficiently advanced^1^,^2^,^3^.

The bacterial divisome is a complex apparatus that contains over a dozen highly-conserved proteins^4^. In *Escherichia coli*, its different components are recruited to the division site in an almost linear pathway^5^,^6^. Two steps can be distinguished. First, a tubulin homologue, FtsZ, polymerises into a cytoplasmic contractile ring structure, the Z-ring, at about 25–38% of the cell cycle^5^,^6^. Several other divisome components, the so-called early cell division proteins, are conjointly recruited with FtsZ. They include FtsA, an actin homologue that anchors the Z-ring to the inner cell membrane and controls the onset of constriction^7^,^8^, and ZapA, which participates to Z-ring stabilisation but also to the recruitment of the replication terminus region of the *E. coli* chromosome to the division site^9^,^10^,^11^,^12^. Second, integral membrane and periplasmic proteins join the Z-ring at about 48–52% of the cell cycle^5^,^6^. These so-called late cell division proteins include FtsK, a bi-functional protein that transports DNA from one daughter cell compartment to another during constriction^13^,^14^,^15^, FtsL, which modulates the onset of cell wall constriction in association with FtsQ and FtsB^16^,^17^, and FtsI (PBP3), a peptidoglycan (PG) transpeptidase that specifically acts at the division site^18^,^19^. The latest divisome component to be recruited is FtsN. It triggers the onset of constriction by changing the conformations of FtsA and FtsQLB^17^,^20^. FtsN contains a periplasmic SPOR domain, which stabilizes it at the division site by specifically binding to the denuded glycan strands that accumulate during constriction in *E. coli* and heterologous hosts^21^,^22^.

The position and timing of assembly of the divisome is coordinated with chromosome segregation. In *E. coli*, this is achieved by the Min and Nucleoid Occlusion (NO) systems, which negatively regulate FtsZ polymerisation^23^,^24^. Of these, Min is considered the most significant. In its absence, Z-rings assemble at places other than mid-cell leading to division events near the cell poles and the formation of anucleate mini-cells^24^. NO is mainly effected by SlmA, a protein that binds to specific DNA motifs that are repeated on the *E. coli* genome, the SlmA Binding Sites (SBSs)^23^,^25^. The role of SlmA becomes apparent only when problems arise during DNA replication or segregation, or in the absence of Min^23^.

The agent of the cholera, *Vibrio cholerae*, is a polarized comma-shaped Gram-negative bacterium. Its genome is divided between a 3 Mbp primary chromosome, Chr1, and a 1 Mbp plasmid-derived chromosome, Chr2^26^. Chr1 and Chr2 are longitudinally arranged within the cell^27^. Their spatial arrangement and choreography of segregation are at least in part dictated by two distinct partition systems, ParA1/ParB1/*parS1* and ParA2/ParB2/*parS2*, and by a single MatP/*matS* system^15^,^28^,^29^. A cell pole protein, HubP, recruits ParA1 to the cell poles, which in turn tethers sister copies of the replication origin region of Chr1, *oriC1*, to the poles^27^,^30^. The second partition system promotes the recruitment of sister copies of the replication origin region of Chr2 to the 1/4 and 3/4 positions^27^,^29^. MatP helps maintain the terminus region of the two chromosomes at mid-cell until the onset of septation^15^. The genome of *V. cholerae* encodes for putative homologues of the large majority of *E. coli* cell division proteins, including FtsZ, FtsA, ZapA, FtsK, FtsI, FtsN, Min and SlmA. SlmA is the main regulator of cell division in *V. cholerae* and the role of Min is only apparent when chromosome organisation is altered^1^. We recently showed that the distribution of SBSs on Chr1 and Chr2 confined FtsZ to the new cell pole of newborn cells and seemed to delay Z-ring assembly to a very late stage of the cell cycle, after most of Chr1 and Chr2 has been replicated and segregated^1^. Thus, recruitment of late cell division proteins and actual septation is restricted to a relatively short portion of the cell cycle in *V. cholerae*.

Here, we investigated the dynamics of formation of the *V. cholerae* divisome using fluorescence microscopy, temperature sensitive mutants and a chemical inhibitor of FtsI. Our results indicate that FtsZ polymerisation presides over the recruitment of the other cell division components, which occurs in two distinct steps. They further suggest that early pre-divisional Z-rings form between 40 and 50% of the cell cycle. Pre-divisional Z-rings evolve into mature divisome at about 80% of the cell cycle when late cell division proteins are recruited. Finally, our results suggest that late divisome assembly participates to the asymmetric polar organisation of *V. cholerae* cells by limiting the accumulation of a cell pole marker, HubP, at the nascent cell poles.

## Results

### The *V. cholerae* divisome forms in two steps

We engineered fluorescent protein fusions of the *V. cholerae* homologues of three early (FtsZ, FtsA and ZapA) and four late (FtsK, FtsL, FtsI and FtsN) *E. coli* cell division proteins. Among these, we obtained evidence that the FtsZ red fluorescent protein C-terminal fusion (FtsZ-RFPT) was partially functional^1^, and that the FtsK green fluorescent protein C-terminal (FtsK-YGFP) and FtsI green fluorescent protein N-terminal (YGFP-FtsI) fusions were fully functional (see^1^ for FtsK-YGFP and Supplementary Figure 1 for YGFP-FtsI). With the exception of FtsK-YGFP, which was introduced in place of the wild-type FtsK ORF at the *ftsK* locus, fluorescent fusions were produced from an ectopic chromosomal locus in presence of the untagged wild-type copy.

Microscopic fluorescence snapshot images revealed that the seven cell division protein fusions targeted mid-cell at the time of cell division (Supplementary Figure 2a). In addition, they formed discrete bands at positions where the divisome machinery was expected to assemble in cell filaments induced by cephalexin, an antibiotic that blocks cell wall constriction but does not stop divisome formation nor the replication and segregation cycle of the genetic material (Supplementary Figure 2b). These results suggested that *V. cholerae* FtsZ, FtsA, ZapA, FtsK, FtsL, FtsI and FtsN were true orthologs of the *E. coli* cell division proteins.

**Figure 1:**
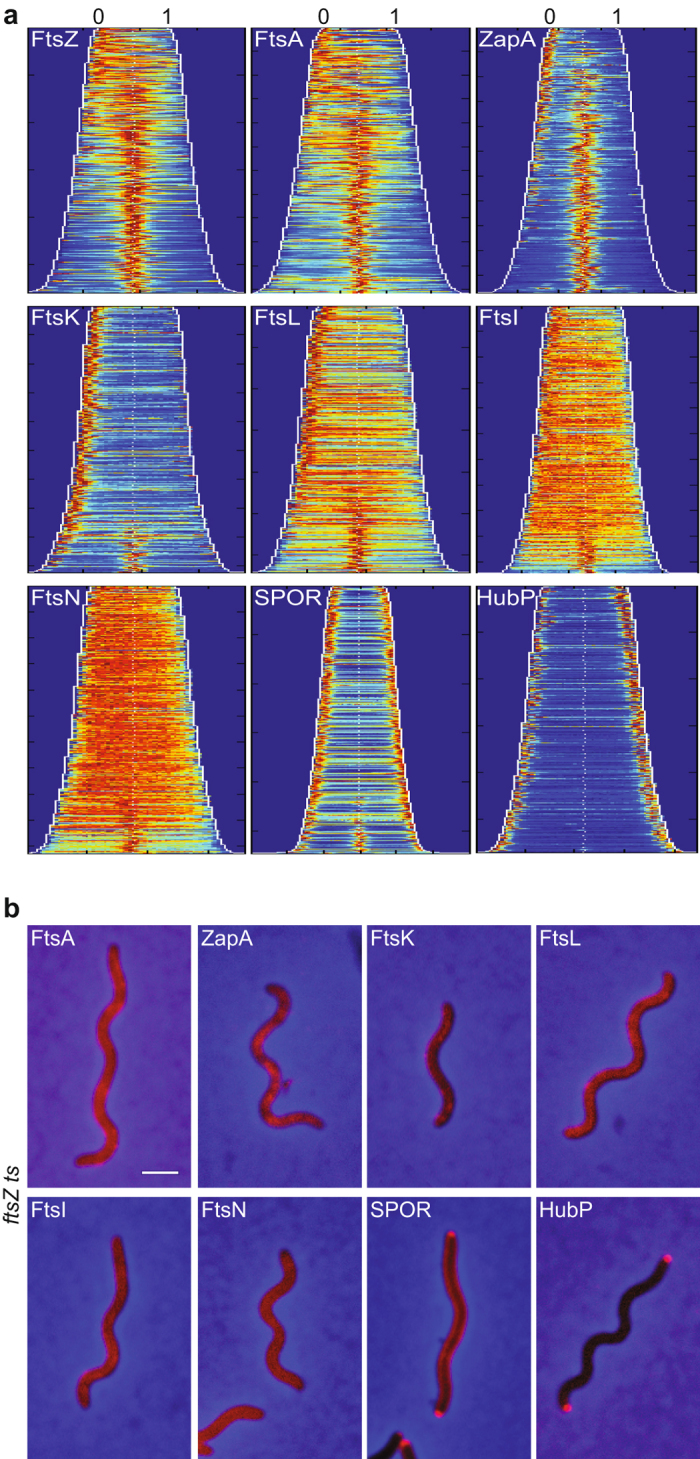
Localisation of *V. cholerae* cell division machinery components. (**a**) Fluorescence profiles (demographs) of *V. cholerae* N16961 cells carrying fluorescently tagged versions of FtsZ (n = 3030), FtsA (n = 2325), ZapA (n = 2570), FtsK (n = 1784), FtsL (n = 3264), FtsI (n = 2182), FtsN (n = 2217), SPOR domain (n = 2768) and HubP (n = 2949). On the y-axis, cells are sorted for length, ascending from top (the shortest cell) to bottom (the longest cell). On the x-axis, 1 corresponds to the old pole and 0 to the new pole. FtsZ, FtsA, ZapA, FtsK, FtsL, FtsI and FtsN are oriented towards the new pole, using the brighter pole as a marker. SPOR domain and HubP are not oriented. In the heat maps blue corresponds to low and dark red to high intensity. (**b**) Cellular localisation of YGFP-FtsA, RFPT-ZapA, FtsK-YGFP, YGFP-FtsL, sfGFP-FtsI, sfGFP-FtsN, DsbA_ss_-mCherry-SPOR and HubP-sfGFP in *V. cholerae ftsZ*^*ts*^ cells after 1 h 30 min incubation at 42 °C.

We plotted demographic representations of the localisation of the seven cell division fluorescent protein fusions as function of cell length (Fig. 1a). Each cell was divided into small sectors perpendicular to the long cell axis and the maximal fluorescence intensity of each sector was projected along the long cell axis to create a long cell axis fluorescence distribution profile. Individual profiles were plotted as horizontal jet colour bars, with dark blue and dark red corresponding to the regions of lowest and highest fluorescence intensity, respectively. In snapshots images, it is not possible to distinguish the new pole, which derives from the most recent septation event, from the old pole. However, we previously showed that *V. cholerae* FtsZ and FtsK remained localised at the new pole after division^1^. Based on this observation, we oriented each fluorescence intensity profile by placing the pole showing the highest fluorescence intensity at the zero position on the x-axis. Profile colour bars were ordered as a function of cell length and piled up in a single image, with the shortest cells at the top of the y-axis and the longest at the bottom.

FtsZ, FtsA and ZapA fluorescent fusions displayed similar demographic maps. In a third of the cells, the shortest ones, fluorescence was maximal at the supposedly new cell pole. In the rest of the cells, it reached its maximum at mid-cell (Fig. 1a, top panels). In contrast, FtsK-YGFP fluorescence remained maximal at the new cell pole in the majority of cells and only re-located to mid-cell in a fifth of the cells, the longest ones (Fig. 1a, middle left panel). A similar pattern could be distinguished for FtsL, FtsI or FtsN fluorescent fusions, despite a very poor signal to noise ratio (Fig. 1a, middle panels and bottom left panel).

We included in our study a fluorescent protein fusion to the *E. coli* FtsN periplasmic SPOR domain as a marker for actual septation. Because the SPOR domain is periplasmic, we used the DsbA signal sequence to efficiently export an N-terminal fusion of the SPOR domain to mCherry (DsbA_ss-_mCherry-SPOR) into the periplasm^31^. Cells producing mCherry-SPOR presented a weak signal along the entire cell membrane, which was enriched at both poles and accumulated at mid-cell in the longest cells (bottom middle panel of Fig. 1a and Supplementary Figure 2a).

Finally, we imaged cells producing a C-terminal fusion of HubP to super folder GFP (HubP-sfGFP) as a marker of new pole formation. HubP-sfGFP complemented the cell division mini-cell defect of a *hubP* deletion mutant in the absence of *minCD*, suggesting that it was fully functional (Supplementary Figure 2c). Previous HubP fluorescent protein fusions were described to localise at both cell poles throughout the cell cycle and to mid-cell at the end of the division process^30^. Correspondingly, we observed fluorescence signals at the two cell poles and at mid-cell in the longest cells when HubP-YFP was produced from an ectopic locus in addition to the endogenous HubP (Supplementary Figure 2d,e). However, when HubP-sfGFP was produced in place of the endogenous HubP, from the native *hubP* locus and under the native *hubP* promoter, signals were only detected at cell poles (bottom right panel of Fig. 1a and Supplementary Figure 2a). In addition, a single pole was labelled in many cells (Supplementary Figure 2a, bottom right panel). Not knowing to which pole it corresponded, we did not orientate the HubP-sfGFP distribution profiles in the demographic representation (bottom right panel of Fig. 1a).

Taken together, these results suggest that the formation of the *V. cholerae* divisome is a two-step process, with FtsZ, FtsA and ZapA assembling into a mid-cell complex earlier than FtsK, FtsL, FtsI and FtsN.

### FtsZ presides over divisome formation

We constructed a *V. cholerae ftsZ* temperature-sensitive (*ftsZ*^*ts*^) strain equivalent to *E. coli ftsZ84* conditional mutant^1^. Cells carrying the *ftsZ*^*ts*^ allele grew at 30 °C, but were unable to proliferate at 42 °C (Supplementary Figure 1a). Microscopy inspection showed that they formed long filaments at 42 °C (Supplementary Figure 1b). All of the proteins we analysed failed to localise at discrete positions along the cell filaments at the non-permissive temperature, except for the SPOR domain and HubP, which still formed bright foci at the cell poles (Fig. 1b). Taken together, these data demonstrate that FtsZ plays an essential primary role for the recruitment of the other cell division proteins at the division site in *V. cholerae*.

### Cell cycle choreography of the cell division proteins

As cell length is related to cell cycle advancement, demographic representations can provide an indirect view of the cell cycle choreography of proteins. Note, however, that the relationship between the y-axis of demographic representations and cell cycle advancement is not linear. As previously described for other rod bacteria^32^,^33^, cell length increases exponentially during the cell cycle in *V. cholerae* (Supplementary Figure 3a,b). In addition, the y-axis does not correspond to cell length but to the ranking number of cells when ordered according to length (Supplementary Figure 3c,e). Taking into account both factors, we estimated that early cell division proteins formed pre-divisional Z-rings between 40 and 50% of the cell cycle (Supplementary Figure 3d) and that late cell division proteins joined them at about 80% of the cell cycle (Supplementary Figure 3f).

To gain a direct view of the cell cycle choreography of cell division proteins, we used time-lapse fluorescent microscopy of micro-colonies grown on agar pads. At each time point, cell contours were determined based on brightfield images and the history of each cell was reconstituted^1^. We plotted individual jet colour image representations of maximal fluorescence intensity along cell axis projections as a function of cell cycle progression for cells for which both birth and division were observed. Observation of the mother cell division event permitted to determine the new and old pole of each cell. Individual images were compiled to create consensus maps of long cell axis fluorescence projection as a function of relative cell cycle progression. In a first set of maps, dark red and blue colours were assigned to the maximal and minimal fluorescence intensity projections observed at each time point (Intensities Scaled at each time point, IS^time^). The IS^time^ representation highlights changes in the relative distribution of fluorescence along the long cell axis. In a second set of maps, dark red and blue colours were assigned to the maximal and minimal fluorescence projection intensities observed over the entire cell cycle (Intensities Scaled over the cell cycle, IS^cycle^). The IS^cycle^ representation highlights stages of the cell cycle in which fluorescence signals are located at the same position for all of the different observed cell lineages.

**Figure 2:**
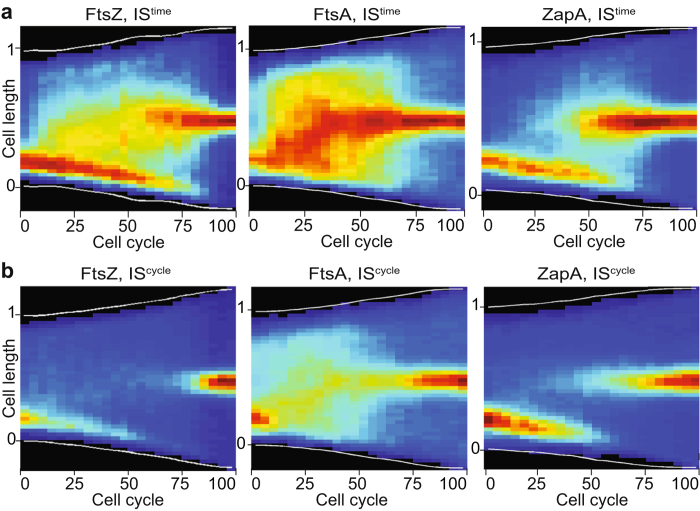
Dynamic localisation of early cell division proteins. Cell cycle distribution of FtsZ-RFPT (compilation of 50 single cell cycles), YGFP-FtsA (compilation of 36 single cell cycles) and RFPT-ZapA (compilation of 55 single cell cycles) in *V. cholerae* N16961 cells. Y-axis: position along the cell length, with 0 corresponding to the new pole and 1 to the old pole. X-axis: cell cycle. (**a**) Heat maps using the IS^time^ representation. (**b**) Heat maps using the IS^cycle^ representation.

Both IS^time^ and IS^cycle^ maps suggested that FtsZ, FtsA and ZapA fluorescent fusions stably located at the new pole of newborn cells (Fig. 2a,b). Comparison of consensus (Fig. 2a) and individual (Supplementary Figure 4a,b) IS^time^ maps further suggested that FtsZ and FtsA molecules were very dynamic and travelled all over the cell before relocating at mid-cell at about 50% of the cell cycle. In contrast, ZapA seemed to directly relocate at mid-cell (Fig. 2b and Supplementary Figure 4c). In the consensus IS^cycle^ map, the FtsZ signal progressively faded from the new pole before reappearing at mid-cell at about 80% of the cell cycle (Fig. 2b). A similar pattern was observed in individual IS^cycle^ maps (Supplementary Figure 4a). These results suggested that (i) FtsZ molecules travelled independently from each other and (ii) that early pre-divisional Z-rings were more loosed and/or less compact than late divisional Z-rings, observed at about 80% of the cell cycle (Movie 1). FtsA and ZapA IS^cycle^ maps were roughly equivalent to FtsZ IS^cycle^ maps (Fig. 2b and Supplementary Figure 4a,b,c). The track of FtsA signal in the transition period between the new pole and mid-cell of the consensus IS^cycle^ map didn’t correspond to any clear track in the individual IS^cycle^ maps (Fig. 2b and Supplementary Figure 4b). Together, these results suggested that FtsA and ZapA had the same behaviour as FtsZ molecules (Fig. 2b and Supplementary Figure 4b,c).

**Figure 3:**
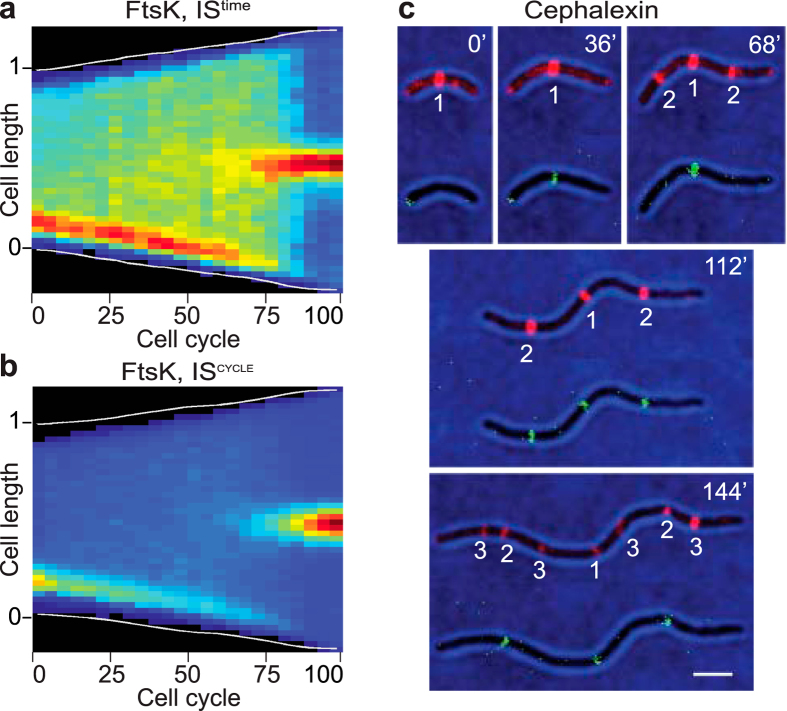
Dynamic localisation of late cell division proteins. (**a**) IS^time^ cell cycle distribution of YGFP-FtsK (compilation of 44 single cell cycles). (**b**) IS^cycle^ cell cycle distribution of DsbA_ss_-mCherry-SPOR (compilation of 44 single cell cycles). (**c**) Time-lapse images of N16961 cells expressing FtsZ-RFPT and FtsK-YGFP in presence of cephalexin. One frame was taken every 4 minutes. Numbers in white indicate the division rounds. On the top-right corner of each frame is indicated the time in minutes from the beginning of imaging. Scale bar = 2 μm.

Among the late cell division proteins, only FtsK could be followed throughout complete division cycles in time-lapse experiments. At birth, all cells displayed an FtsK signal at the new pole (Fig. 3a,b and Supplementary Figure 4d). The new pole signal remained visible until 75% of the cell cycle and a mid-cell signal appeared only after 70% of the cell cycle had elapsed (Fig. 3a,b and Supplementary Figure 4d). To further analyse the recruitment of FtsK at division sites, we followed fluorescent protein fusions of FtsZ and FtsK in cephalexin treated cells using dual-colour time-lapse microscopy. Cephalexin-treatment does not stop the replication and segregation cycle of the genetic material and each round of replication/segregation leads to the assembly of new divisomes in the filaments: a single divisome forms at mid-cell in the first round; 2 new divisomes form at 1/4 and 3/4 of the cell length in the second round; 4 new divisomes form at 1/8, 3/8, 5/8 and 7/8 of the cell length in the third round (Fig. 3c). FtsZ fluorescent fusion signals invariably located prior to FtsK fluorescent fusion signals at the division sites, confirming that formation of the *V. cholerae* divisome is a two-step process (Fig. 3c and Movie 2). Taken together, these results clearly demarked FtsK as a late cell division protein compared to FtsZ, FtsA and ZapA, which confirmed that divisome formation is a two-step process. Finally, the FtsK-YGFP IS^cycle^ map suggested that FtsK formed a stable mid-cell complex at 80% of the cell cycle, almost concomitantly with the formation of a stable compact late divisional Z-ring (Fig. 3b and Supplementary Figure 4d).

### Cell wall constriction initiates in the last 10% of the cell cycle

**Figure 4:**
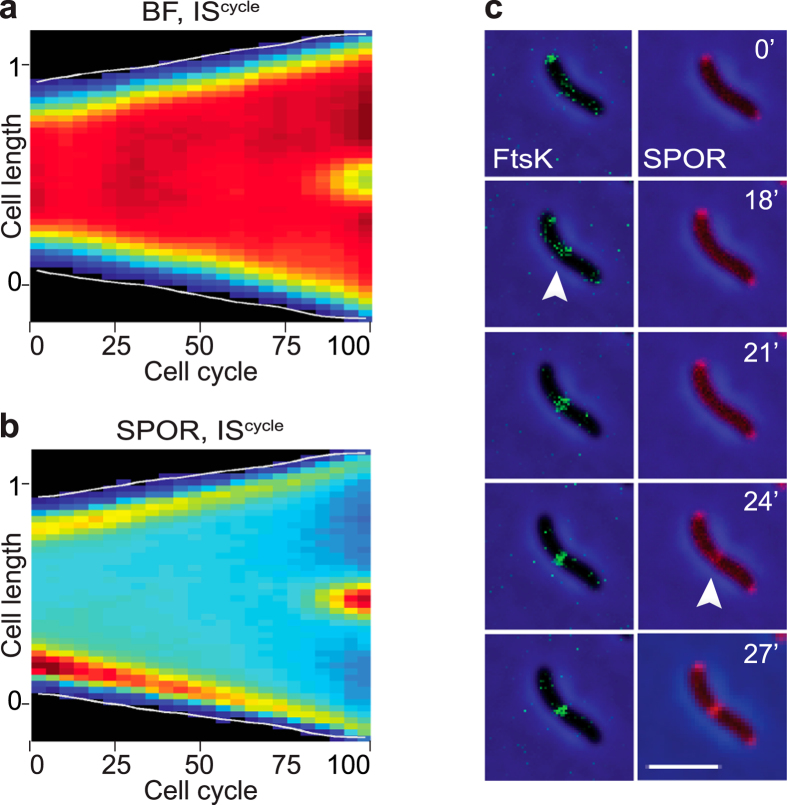
Late initiation of cell wall constriction. (**a**) IS^cycle^ cell cycle distribution of BF images (compilation of 68 single cell cycles). (**b**) IS^cycle^ cell cycle distribution of DsbA_ss_-mCherry-SPOR (compilation of 44 single cell cycles). (**c**) Time-lapse images of N16961 cells expressing YGFP-FtsK and DsbA_ss_-mCherry-SPOR. One frame was taken every 3 minutes. On the top-right corner of each frame is indicated the time in minutes from the beginning of imaging. White arrows show the arrival of tagged proteins to mid-cell. Scale bar = 2 μm.

We next investigated the time at which actual septation initiated. Cell constriction leads to an increase in the transparency of the division site, which can be directly observed in brightfield images. The brightfield IS^cycle^ map suggested that constriction started at 90% of the cell cycle (Fig. 4a). To confirm this observation, we followed the choreography of a SPOR domain fluorescent fusion using time-lapse microscopy as a mark for the formation of new denuded glycan. IS^cycle^ and IS^time^ maps were almost identical (Fig. 4b, Supplementary Figure 4e and Supplementary Figure 5a). They suggested that the SPOR domain located at both poles in newborn cells (Fig. 4b). However, fluorescence intensity was stronger at the new pole (Fig. 4b). In this regard, the choreography of the SPOR domain resembled the choreography of all other examined divisome components. Indeed, the IS^time^ map of the SPOR domain suggested that it started to relocate at the division site when both FtsZ and FtsK signals were maximal (Supplementary Figure 5a). However, the IS^cycle^ map showed that the SPOR signal only reached its maximal intensity at a later time, when around 90% of the cell cycle had elapsed (Figs 2b and 3b). To further refine when new peptidoglycan started to be incorporated at the septum, we compared the arrival of the SPOR domain with respect to the arrival of FtsK using dual-colour time-lapse fluorescent microscopy. FtsK invariably reached mid-cell ahead of the SPOR domain, confirming that there is a delay between the final assembly of the divisome and its maturation into an active cell wall remodelling machinery (Fig. 4c and Supplementary Figure 5b).

### HubP leads Chr1 segregation

**Figure 5:**
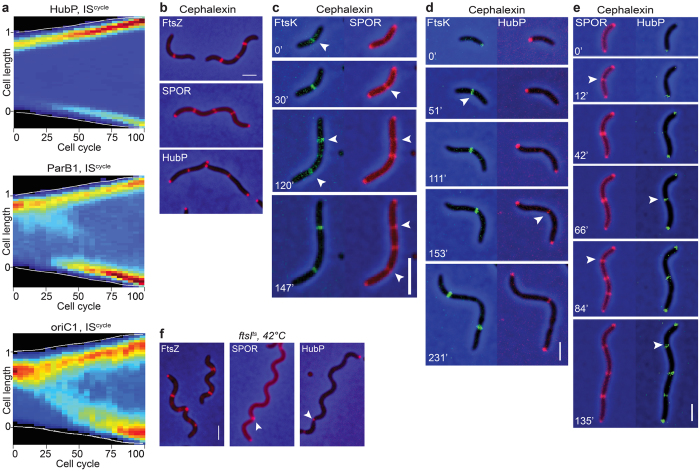
Recruitment to the divisome in 4 temporally differentiated phases. (**a**) IS^cycle^ cell cycle distribution of HubP-sfGFP (compilation of 88 single cell cycles), YGFP-ParB1 (compilation of 69 single cell cycles) and *oriC1* locus (compilation of 50 single cell cycles). (**b**) Cellular localisation of FtsZ-RFPT, DsbA_ss_-mCherry-SPOR and HubP-sfGFP in cephalexin treated cells. (**c,d,e**). Time-lapse images of N16961 cells expressing (**c**) FtsK-YGFP and DsbA_ss_-mCherry-SPOR, (**d**) FtsK-YGFP and HubP-RFPT, (**e**) DsbA_ss_-mCherry-SPOR and HubP-sfGFP in presence of cephalexin. One frame was taken every 4 minutes. On the top-right corner of each frame is indicated the time in minutes from the beginning of imaging. White arrows show the arrival of tagged proteins to putative division sites. (**f**) Cellular localisation of FtsZ-RFPT, DsbA_ss_-mCherry-SPOR and HubP-sfGFP in *V. cholerae ftsI*^*ts*^ cells after 1 h 30 min incubation at 42 °C. Scale bar = 2 μm.

Snapshot images of HubP suggested that it localized at a single pole in newborn cells. Time-lapse experiments further indicated that it localised at the old pole of newborn cells and gradually accumulated at the opposite pole (Fig. 5a, Supplementary Figures 6a and 7a). The polar localisation pattern of the HubP signal was opposite to the signal localisation pattern of late cell division proteins. However, the FtsK choreography of Δ*hubP* cells did not appear different from that of wild-type cells, suggesting that HubP was not actively involved in signalling when late cell division proteins have to leave the new pole (Supplementary Figure 6b).

HubP has been described to be necessary for the proper localisation of the origin of replication of Chr1, *oriC1*, to the poles through an interaction with ParA1^30^. We decided therefore to explore the cell cycle movement of *oriC1* by inserting a *lacO* array in its proximity and expressing a LacI-YGFP fusion inserted at *lacZ*. In addition, we followed the cell cycle localisation of the origin-associated centromere-binding protein ParB1, which specifically binds to *parS1* original-proximal sites and is targeted to the poles by ParA1 during segregation^28^. Both ParB1 and LacI fluorescent fusions located at the old pole at birth, but a second copy of them reached the opposite pole at 45 and 70% of the cell cycle, respectively (Fig. 5a, Supplementary Figures 6a and 7b,c). In the case of ParB1 and LacI-YGFP, it was possible to detect a track moving from the old pole towards the new pole (Fig. 5a and Supplementary Figure 6a). The tracks were also observed in individual IS^time^ and IS^cycle^ maps, suggesting that they corresponded to molecules travelling in the form of a complex (Supplementary Figure 7b,c). Such a distinct track could not be observed for the HubP fluorescent fusion (Figs 5a, Supplementary 6a and 7a).

Taken together, these results suggested that HubP reached the new pole first, shortly followed by a complex of ParB1 molecules and lastly by one of the two duplicated sister *oriC1* loci.

### Delayed septation permits to recruit HubP to division sites

Both HubP and the SPOR domain fluorescent fusions formed bright foci at the poles and discrete spatially distributed bands at the expected divisome positions in cephalexin treated cells (Fig. 5b). It allowed us to directly time their arrival at the division sites with respect to the arrival of FtsK. The SPOR domain reached the divisome after FtsK (Fig. 5c and Movie 3). HubP recruitment to the divisome machinery was even further delayed to after the arrival of both FtsK (Fig. 5d and Movie 4) and the SPOR domain (Fig. 5e and Movie 5). Even when overproduced from an arabinose promoter, a fluorescent derivative of HubP reached the nascent poles at a very late stage of septum formation, after the SPOR domain (Supplementary Figure 6c and Movie 6). Taken together these results suggest that HubP recognises a specific feature of the division site and that given sufficient time it is able to target the position of the two future nascent poles before their formation.

### Recruitment of the SPOR domain and of HubP depends on FtsI

Localisation of the SPOR domain at division sites in cephalexin-treated *V. cholerae* cells was particularly surprising because the SPOR domain is delocalized throughout the periplasm in cephalexin-treated *E. coli* cells^34^,^35^. To further characterise the mode of recruitment of the SPOR domain and of HubP at division sites, we engineered a *V. cholerae ftsI*^*ts*^ conditional mutant equivalent to *E. coli ftsI23*^36^ (Supplementary Figure 1). In this background, FtsZ localized at the putative division sites at non-permissive temperature (Fig. 5f). In contrast, both the SPOR domain and HubP were only present at the cell poles and at sites of deep cell wall constrictions (white arrows) (Fig. 5f). Taken together, these results suggest that FtsI is necessary to target the SPOR domain and HubP at future division sites in *V. cholerae*.

## Discussion

The overarching principles driving bacterial cell division and its coordination to the replication and segregation cycle of the genome are broadly conserved: the first step of cell division is generally the formation of a ring-like structure by a tubulin homologue, FtsZ, which is guided by the cellular arrangement of the genomic DNA, the shape of the cell and/or the composition of its lipid and peptidoglycan envelopes. Nevertheless, substantial differences in cell division have been observed in several species, probably as an adaptation to cell shape and cellular organisation specificities and/or to particular developmental programmes and ecological niches. For instance, two FtsZ polymerisation inhibitory systems, Min and NO, guide divisome assembly at mid-cell in *B. subtilis* and *E. coli*^3^. NO blocks Z-ring assembly over the bulk of the nucleoid. Min blocks Z-ring assembly at the cell poles, which chases cell division proteins from the new pole after division. In contrast, a single inhibitor of FtsZ-polymerisation, MipZ, performs both tasks in *C. crescentus*^3^. *C. crescentus* is a curved rod with an old pole to new pole polarity. MipZ localises at the old pole of newborn cells. As a result, cell division proteins remain at the new pole after division in *C. crescentus*^3^. In addition, FtsA, an actin homologue that anchors the Z-ring to the inner cell membrane, is recruited at the division sites at an early stage in *E. coli* and *B. subtilis* and at a late stage in *C. crescentus*^37^.

### The *V. cholerae* divisome assembly pathway follows the *E. coli* paradigm

The genome of *V. cholerae* encodes for homologues of the major components of the *E. coli* divisome machinery, including FtsZ and FtsK, and homologues of the Min and NO division regulatory systems. As in *E. coli*, NO is mainly effected by an FtsZ-polymerisation antagonist, SlmA, which binds to specific DNA motifs, the SlmA Binding Sites (SBSs)^1^. Unlike *E. coli*, however, *V. cholerae* harbours two chromosomes, Chr1 and Chr2, which are longitudinally arranged inside the cell^27^. We recently showed that the genomic distribution of SBSs, combined with the cellular arrangement of the two *V. cholerae* chromosomes, confined FtsZ and FtsK to the new cell pole of newborn cells^1^. In this regard, *V. cholerae* was more akin to *C. crescentus*, another curved polar rod, than to *E. coli*, which called for a more detailed analysis of its cell division process.

**Figure 6:**
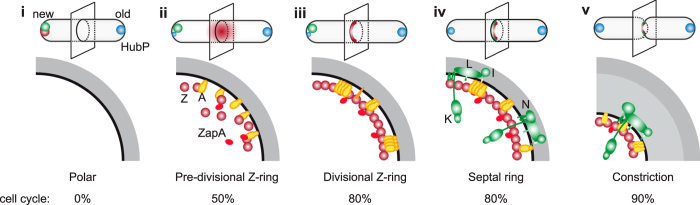
*V. cholerae* divisome assembly. Schematic representation of the divisome assembly process. Top panels show a depiction of the entire cell throughout the cell cycle, bottom panels a magnified view of the mid-cell section. Fts cell division proteins are indicated with the last letter of their name. (i) In newborn cells all cell division proteins localise at the new cell pole; (ii) as cells elongate, early cell division proteins FtsZ, FtsA and ZapA transition to mid-cell whereas late cell division proteins (e.g. FtsK, FtsL, FtsI, FtsN) remain at the new pole (50% of the cell cycle); (iii) formation of tighter Z-ring structures (80% of the cell cycle); (iv) at the end of the cell elongation phase all division proteins are assembled at mid-cell (80% of the cell cycle) and initiation of constriction starts (90% of the cell cycle) (v). HubP is present at the old pole in newborn cells (i) and gradually accumulates at the opposite pole during elongation (ii, iii, iv) until it is equally distributed at both poles at the time of constriction (v).

Here, we followed the choreography of fluorescent protein fusions of five cell division proteins – ZapA, FtsA, FtsL, FtsI and FtsN, in addition to FtsZ and FtsK. They all targeted mid-cell in the longest cells under normal growth conditions and formed discrete bands at positions where the divisome machinery was expected to assemble in cephalexin-induced filaments, suggesting that they were true orthologs of the *E. coli* cell division proteins (Fig. 1 and Supplementary Figure 2). On the basis of the longitudinal distribution of the fluorescence signal as a function of cell length (Fig. 1), the time at which it disappeared from the new pole (Fig. 2 and Fig. 3) and appeared at the new division sites in cephalexin-induced filaments (Fig. 3), the analysed proteins were grouped into the same early and late cell division protein categories as their *E. coli* homologues (Fig. 6). In particular, *V. cholerae* FtsA grouped with the FtsZ and ZapA early cell division proteins in contrast to the *C. crescentus* situation^37^. Finally, we showed that FtsA, ZapA, FtsK, FtsL, FtsI and FtsN all depended on FtsZ for proper localisation at the division sites, suggesting that FtsZ recruitment at mid-cell is the primary event in the cell division process (Fig. 1). Together, these results suggest that the assembly pathway of the *V. cholerae* divisome follows the *E. coli* 2-steps assembly paradigm.

### Maturation of early pre-divisional Z-rings into divisomes is concomitant with the recruitment of late cell division proteins in *V. cholerae*

We used time-lapse fluorescent microscopy to time the different stages of the cell division process. Only FtsZ, FtsA, ZapA and FtsK were bright enough to be followed by time-lapse microscopy (Fig. 2 and Fig. 3). However, snapshot images suggested that FtsL, FtsI and FtsN behaved as FtsK (Fig. 1). We presented the data in two different ways to highlight changes in the relative distribution of the fluorescent protein fusions along the long cell axis (IS^time^) and to determine the portion of the cell cycle when the fluorescent protein fusions assumed a constant position from time point to time point and from cell to cell (IS^cycle^). Taken together our results suggest that all *V. cholerae* divisome components localise at the newly formed pole at birth (Figs 2 and 3, IS^cycle^; Fig. 6,i). However, IS^time^ maps suggest that they do not assemble into a stable structure at this location and that molecules navigate all over the cell (Figs 2 and 3, IS^time^). FtsZ and the early cell division proteins re-localise in a pre-divisional Z-ring at mid-cell at ~50% of the cell cycle (Fig. 2, IS^time^; Fig. 6,ii) but only form a stable tight structure at ~80% of the cell cycle (Fig. 2, IS^cycle^; Fig. 6,iii). Late stabilisation of Z-rings is at least in part explained by the presence of SlmA-bound DNA at mid-cell until a late stage of the cell cycle ^1^. However, FtsK and probably the other late cell division proteins re-locate to mid-cell almost concomitantly with the formation of a stable compact Z-ring (Fig. 3, IS^time^ and IS^cycle^; Fig. 6,iv). Therefore, we cannot exclude that the late cell division proteins play a role in the stabilisation of early pre-divisional Z-rings.

### Actual septation occupies less than 10% of *V. cholerae* cell cycle

Whether in *E. coli, B. subtilis* or *C. crescentus*, the Z-ring forms at a 3^rd^ of the cell cycle, late cell division proteins are recruited at half of the cell cycle, leaving the remaining half of the cell cycle to perform cell wall constriction and cell scission^37^,^38^,^39^. In contrast, septal peptidoglycan synthesis and cell wall invagination, highlighted by the recruitment of the SPOR domain at mid-cell and the direct observation of cellular pinching, initiates at ~90% of the cell cycle in *V. cholerae* (Fig. 4; Fig. 6,v). Thus, complete cell scission, from the initiation of invagination to cell separation, takes only 10% of *V. cholerae* cell cycle. This is all the more surprising, as *V. cholerae* has a shorter generation time than *E. coli, B. subtilis* and *C. crescentus*. What features of the *V. cholerae* cell wall remodelling machinery permit it to complete septation in a short time remains to be elucidated. In this regard, it is interesting to note that cephalexin abolishes the recruitment of the SPOR domain at division sites in *E. coli* but not in *V. cholerae*. It suggests that either cephalexin doesn’t completely stop the cell wall remodelling activity of *V. cholerae* FtsI or that the *V. cholerae* divisome harbours an enzyme that can initiate some peptidoglycan synthesis when FtsI is inactivated.

### HubP is a marker of the old pole

HubP is required for the polar placement of *oriC1* through its interaction with ParA1^30^. In its absence, origin segregation is impaired. However, how HubP could orchestrate the segregation of *oriC1* sister copies remained enigmatic. Indeed, initial work on HubP suggested that it targeted both the old pole and the new pole of the cell and even nascent poles during constriction, whereas ParB1 and *oriC1* were specifically associated to the old pole in newborn cells^30^. Our localisation studies of a fully functional HubP-sfGFP fusion inserted in place of the *hubP* ORF solve this apparent paradox. They suggest that HubP almost exclusively localises at the old pole in newborn cells (Fig. 5; Fig. 6,i) and that during cell elongation it progressively adopts a bipolar localization, with new pole concentration slowly increasing until cell scission (Fig. 5; Fig. 6,ii-v). We observed an almost perfect correspondence between the HubP, ParB1 and *oriC1* choreographies (Fig. 5). The only noticeable difference was that a visible transition path highlighted the migration of ParB1 and *oriC1* towards the opposite pole whereas such a track was clearly absent in the HubP map (Fig. 5). These observations suggest that changes in the global polar distribution of HubP are due to the individual dissociation of HubP molecules from the old pole and their individual re-association to the new pole. In contrast, ParB1 and LacI fluorescent molecules tagging *oriC1* migrate from one pole to the other in the form of complexes.

### HubP recognises a specific feature of future division sites

When overproduced, HubP-sfGFP targeted both the old and the new cell poles (Supplementary Figure 2). It also associated with the nascent poles during constriction (Supplementary Figure 2). In addition, HubP-sfGFP targeted division sites in cephalexin-treated cells, in which septation but not divisome formation is blocked (Fig. 5). Finally, HubP did not localise to division sites in *ftsZ*^*ts*^ and *ftsI*^*ts*^ cells at the non-permissive temperature (Fig. 1 and Fig. 5, respectively). As no indentation is observed in *ftsZ*^*ts*^ and *ftsI*^*ts*^ filaments, these results suggest that HubP does not recognise membrane curvature, as proposed for DivIVA of *B. subtilis*^40^,^41^, but rather recognises specific cell wall structures and/or proteins that are associated to a late stage of division. In this regard, it is interesting to note that HubP harbours a LysM periplasmic domain, which binds to peptidoglycan^42^. Future studies will need to investigate which peculiar features, whether protein or cell wall, differentiate nascent, new and old poles and their link with HubP localisation.

## Materials and Methods

### Plasmids and strains

Bacterial strains and plasmids used in this study are listed in Supplementary Table 1. All strains are derivatives of El Tor N16961 strain rendered competent by the insertion of *hapR* and were constructed by natural transformation. Engineered strains were confirmed by PCR.

The *ftsK-YGFP, hubP-RFPT* and *hubP-sfGFP* fusions were inserted in place of the endogenous *V. cholerae ftsK* and *hubP* alleles, respectively. All other fusion proteins were introduced at the *lacZ* or the *hapR* locus. LacI-YGFP, YGFP-ParB1, sfGFP-FtsN and sfGFP-FtsI fluorescent protein fusions were produced from the *E. coli lacZ* promoter, leakiness of the promoter was sufficient for imaging. FtsZ-RFPT, RFPT-ZapA, YGFP-FtsA, YGFP-FtsL, DsbA_ss_-mCherry-SPOR and HubP-YFP fluorescent protein fusions were produced from the arabinose promoter using 0.02% of L-Arabinose.

### Fluorescence microscopy

Cells were grown in M9 minimal medium supplemented with 0.2% fructose and 1 μg/ml thiamine to exponential phase, yielding a 80 minutes generation time. Cultures were spread on a 1% (w/v) agarose pad (ultrapure agarose, Invitrogen) of the same medium for microscopic analysis. For snapshot analyses, cells images were acquired using a DM6000-B (Leica) microscope and were analysed using MicrobeTracker^43^. In demograph representations, cells could be oriented based on the fluorescent signal. For time-lapse analyses, the slides were incubated at 30 °C and images acquired using an Evolve 512 EMCCD camera (Roper Scientific) attached to an Axio Observe spinning disk (Zeiss). FtsI was inhibited by the addition of cephalexin (10 μg/ml, final concentration) directly to the agarose slide. At each time point, we took a stack of 32 brightfield images covering positions 1.6 μm below and above the focal plane. Cell contours were detected and cell genealogies were retraced with a MatLab-based script developed in the lab^1^. After the first division event, the new pole and old pole of the cells could be unambiguously attributed based on the previous division events. Cell cycle choreography heat maps are represented in two different ways: in the IS^time^ representation dark blue corresponds to low and dark red to high intensities, considering every single time-frame of the time-lapse as an independent image whereas in the IS^cycle^ representation the heat maps are calculated using the maximum of fluorescence intensity during an entire cell cycle. The generation time of *V. cholerae* strains during time-lapse experiments was around 80 minutes.

## Additional Information

**How to cite this article:** Galli, E. *et al*. Late assembly of the *Vibrio cholerae* cell division machinery postpones septation to the last 10% of the cell cycle. *Sci. Rep.* **7**, 44505; doi: 10.1038/srep44505 (2017).

**Publisher's note:** Springer Nature remains neutral with regard to jurisdictional claims in published maps and institutional affiliations.

## Supplementary Material

Supplementary Information

Supplementary Movie 1

Supplementary Movie 2

Supplementary Movie 3

Supplementary Movie 4

Supplementary Movie 5

Supplementary Movie 6
