# Supplementary material for: Late assembly of the Vibrio cholerae cell division machinery postpones septation to the last 10% of the cell cycle
Source: Sci Rep. 2017 Mar 16;7:44505. doi: 10.1038/srep44505 (PMC5353653; doi:10.1038/srep44505)
Supplement: Supplementary Information [file srep44505-s1.pdf]

**Late assembly of the *Vibrio cholerae* cell division machinery postpones septation to the last 10% of the cell cycle**

Elisa Galli [1], Evelyne Paly [1] and François-Xavier Barre [1,#]

[1] Institute for Integrative Biology of the Cell (I2BC), Université Paris-Saclay, CEA, CNRS, Université Paris Sud, France

**Supplementary Figure 1. a.** Cells were grown overnight in LB at 30°C. After normalization for cell density, each culture was serially diluted. 5 µl of each dilution was spotted on a LB plate incubated overnight either at 30°C or at 42°C and photographed. **b.** Phase-contrast images of *V. cholerae* N16961 cells carrying the *ftsZ*$^{ts}$ or *ftsI*$^{ts}$ allele after 1h and 30min growth at 42°C. **c.** Combined phase-contrast and fluorescent image of *ftsI*$^{ts}$ *V. cholerae* cells ectopically expressing YGFP-FtsI at 42°C. Scale bar = 2 µm.

**Supplementary Figure 2. a.** Cellular localisation of FtsZ-RFPT, YGFP-FtsA, RFPT-ZapA, FtsK-YGFP, YGFP-FtsL, sfGFP-FtsI, sfGFP-FtsN, $DsbA_{SS}$-mCherry-SPOR and HubP-sfGFP in *V. cholerae* N16961 cells. Scale bar = 2 µm. **b.** Phase-contrast image and cellular localisation of YGFP-FtsA, RFPT-ZapA, FtsK-YGFP, YGFP-FtsL, sfGFP-FtsI and sfGFP-FtsN in cephalexin treated cells. Scale bar = 2 µm. **c.** Percentage of minicells formed at each division in LB as determined from 12 independent time-lapse experiments. *hubP-sfGFP* is integrated at *hubP* native locus and expressed from the native promoter. Error bars represent standard deviation. **d.** Cellular localisation of HubP-YFP ectopically expressed from a $P_{BAD}$ promoter in addition to *hubP* wild-type copy. Scale bar = 2 µm. **e.** Demograph of *V. cholerae* N16961 cells ectopically expressing HubP-YFP from a $P_{BAD}$ promoter in addition to *hubP* wild-type copy. Cells were not oriented.

**Supplementary Figure 3.** Theoretical deduction of the cell cycle advancement of cells using their length. **a.** *V. cholerae* cell length increases exponentially with cell cycle. Log scale representation of cell length as a function of cell cycle advancement for individual *V. cholerae* cells grown on a 1% (w/v) agarose pad in M9 minimal medium, 0.2% fructose and 1 µg/ml thiamine. Blue circles: results for 215 complete lineages, i.e. for cells in which both birth and division were

observed; red line: median log of the length as a function of the cell cycle; black line: linear regression; M: length at division; m: length at birth. **b.** Scheme depicting the relationship between cell cycle advancement and cell length. At 50% of the cell cycle, cell elongation is smaller than half of the total elongation from birth to division. M; division length; m: birth length; (M+m)/2: half of the total elongation from birth to division. **c.** Cell length distribution of 3030 *V. cholerae* cells ectopically producing a fluorescently tagged copy of FtsZ. M: largest observed cell length; m: smallest observed cell length. Marks showing the theoretical cell cycle stages are shown as black (0.1-0.4, 0.6-0.9) and red (0,0.25, 0.5,0.75,1) ticks. Tick positions were determined using the exponential relationship between cell length and cell cycle and assuming that M and m correspond to the division length and the birth length. **d.** FtsZ-localisation in demograph representation. Marks showing the theoretical cell cycle stages are shown as black (0.2-0.4, 0.6, 0.8) and red (0,0.25,0.5,0.75,1) ticks. Each cell cycle stage tick is located at the projection profile of the longest cell whose length is below the theoretical length reached at this particular stage. **e.** Cell length distribution of 1784 *V. cholerae* cells ectopically producing a fluorescently tagged copy of FtsK. M: largest observed cell length; m: smallest observed cell length. Marks showing the theoretical cell cycle stages are shown as black (0.1-0.4, 0.6-0.9) and red (0,0.25,0.5,0.75,1) ticks. Tick positions were determined using the exponential relationship between cell length and cell cycle and assuming that M and m correspond to the division length and the birth length. **f.** FtsK-localisation in demograph representation. Marks showing the theoretical cell cycle stages are shown as black (0.2-0.4, 0.6, 0.8) and red (0,0.25,0.5,0.75,1) ticks. Each cell cycle stage tick is located at the projection profile of the longest cell whose length is

below the theoretical length reached at this particular stage.

**Supplementary Figure 4.** Examples of time-lapse individual lineages in the $\text{IS}^{\text{time}}$ and $\text{IS}^{\text{cycle}}$ representation. O, old pole; N, new pole. **a.** FtsZ-RFPT. **b.** YGFP-FtsA. **c.** RFPT-ZapA. **d.** FtsK-YGFP. **e.** $\text{DsbA}_{\text{SS}}$-mCherry-SPOR.

**Supplementary Figure 5. a.** $\text{IS}^{\text{time}}$ cell cycle distribution of $\text{DsbA}_{\text{SS}}$-mCherry-SPOR (compilation of 44 single cell cycles). **b.** Examples (i, ii) of time-lapse images of N16961 cells expressing YGFP-FtsK and $\text{DsbA}_{\text{SS}}$-mCherry-SPOR. One frame was taken every 3 minutes. On the top-right corner of each frame is indicated the time in minutes from the beginning of imaging. White arrows show the arrival of tagged proteins to mid-cell. Scale bar = 2 µm.

**Supplementary Figure 6. a.** $\text{IS}^{\text{time}}$ cell cycle distribution of HubP-sfGFP (compilation of 88 single cell cycles), YGFP-ParB1 (compilation of 69 single cell cycles) and *oriC1* locus (compilation of 50 single cell cycles). **b.** $\text{IS}^{\text{cycle}}$ cell cycle distribution of YGFP-FtsK (compilation of 73 single cell cycles) in *V. cholerae* N16961 Δ*hubP* cells. **c.** Time-lapse images of N16961 cells expressing $\text{DsbA}_{\text{SS}}$-mCherry-SPOR and HubP-YFP, ectopically expressed from a $\text{P}_{\text{BAD}}$ promoter in addition to *hubP* wild-type copy. One frame was taken every 4 minutes. On the top-right corner of each frame is indicated the time in minutes from the beginning of imaging. White arrows mark proteins arrival at the division sites. Scale bar = 2 µm.

**Supplementary Figure 7.** Examples of time-lapse individual lineages in the $\text{IS}^{\text{time}}$ and $\text{IS}^{\text{cycle}}$ representation. O, old pole; N, new pole. **a.** HubP-sfGFP. **b.** YGFP-ParB1. **c.** Movement of *oriC1* followed by inserting a *lacO* array in its proximity and expressing a LacI-YGFP fusion.

**Supplementary Table 1.** List of strains and plasmids used in this study.

**Movie 1.** Examples of FtsZ-RFPT time-lapses in *V. cholerae* cells. One frame was taken every 3 minutes. Cells were grown in M9 minimal medium supplemented with 0.2% fructose and 1 µg/ml thiamine at 30°C. Movies start from the beginning of imaging.

**Movie 2.** Time-lapse of FtsZ-RFPT and FtsK-YGFP localisation in *V. cholerae* cells. One frame was taken every 4 minutes. Cells were grown in M9 minimal medium supplemented with 0.2% fructose and 1 µg/ml thiamine at 30°C. 10 µg/ml cephalexin was added to the agarose slide.

**Movie 3.** Time-lapse of FtsK-YGFP and $DsbA_{SS}$-mCherry-SPOR localisation in *V. cholerae* cells. One frame was taken every 3 minutes. Cells were grown in M9 minimal medium supplemented with 0.2% fructose and 1 µg/ml thiamine at 30°C. 10 µg/ml cephalexin was added to the agarose slide.

**Movie 4.** Time-lapse of FtsK-YGFP and HubP-RFPT localisation in *V. cholerae* cells. One frame was taken every 3 minutes. Cells were grown in M9 minimal medium supplemented with 0.2% fructose and 1 µg/ml thiamine at 30°C. 10 µg/ml cephalexin was added to the agarose slide.

**Movie 5.** Time-lapse of $DsbA_{SS}$-mCherry-SPOR and HubP-sfGFP localisation in *V. cholerae* cells. One frame was taken every 3 minutes. Cells were grown in M9 minimal medium supplemented with 0.2% fructose and 1 µg/ml thiamine at 30°C. 10 µg/ml cephalexin was added to the agarose slide.

**Movie 6.** Time-lapse of $DsbA_{SS}$-mCherry-SPOR and HubP-YFP localisation in *V. cholerae* cells. HubP-YFP was ectopically expressed from a $P_{BAD}$ promoter in addition to *hubP* wild-type copy. One frame was taken every 4 minutes. Cells were grown in M9 minimal medium supplemented with 0.2% fructose and 1 µg/ml thiamine at 30°C. 10 µg/ml cephalexin was added to the agarose slide.

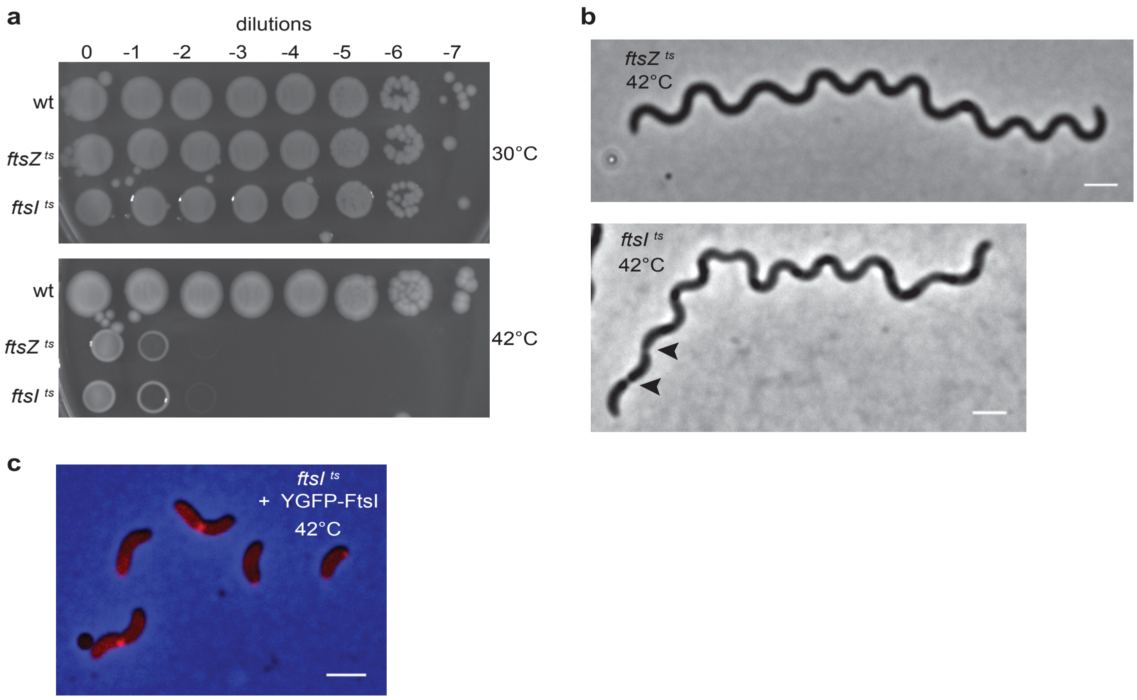


Supplementary Figure 1

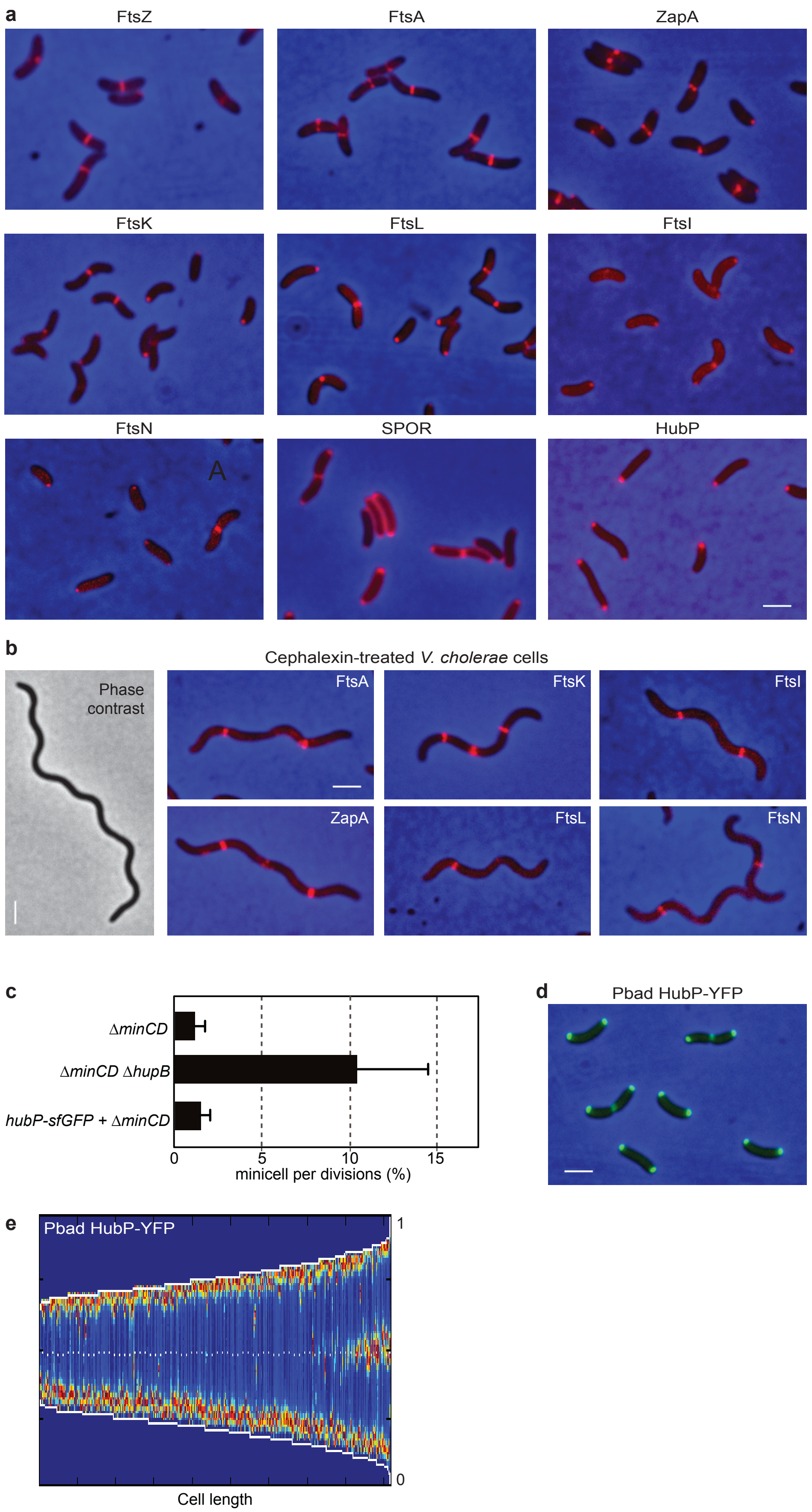


Supplementary Figure 2

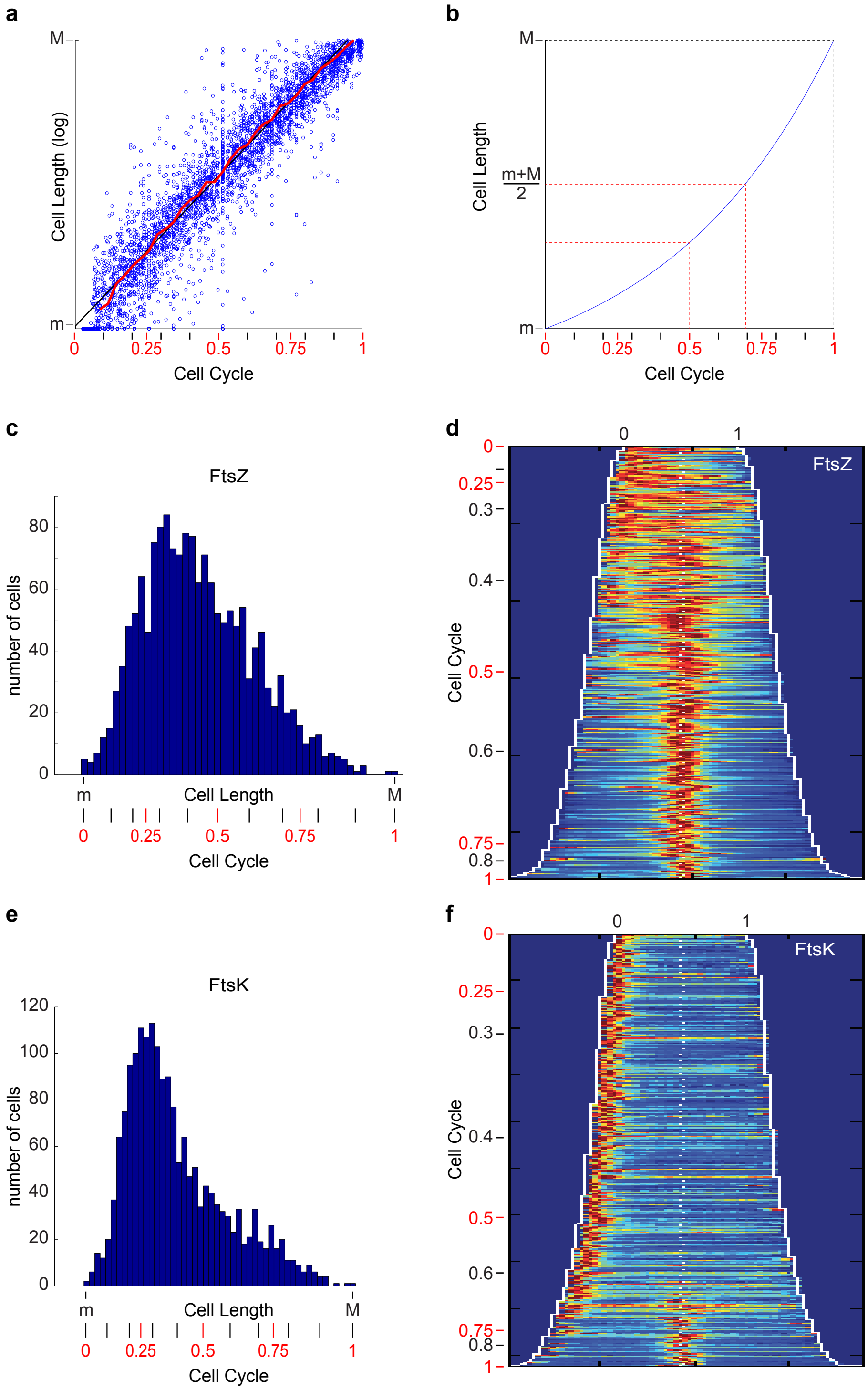


Supplementary Figure 3

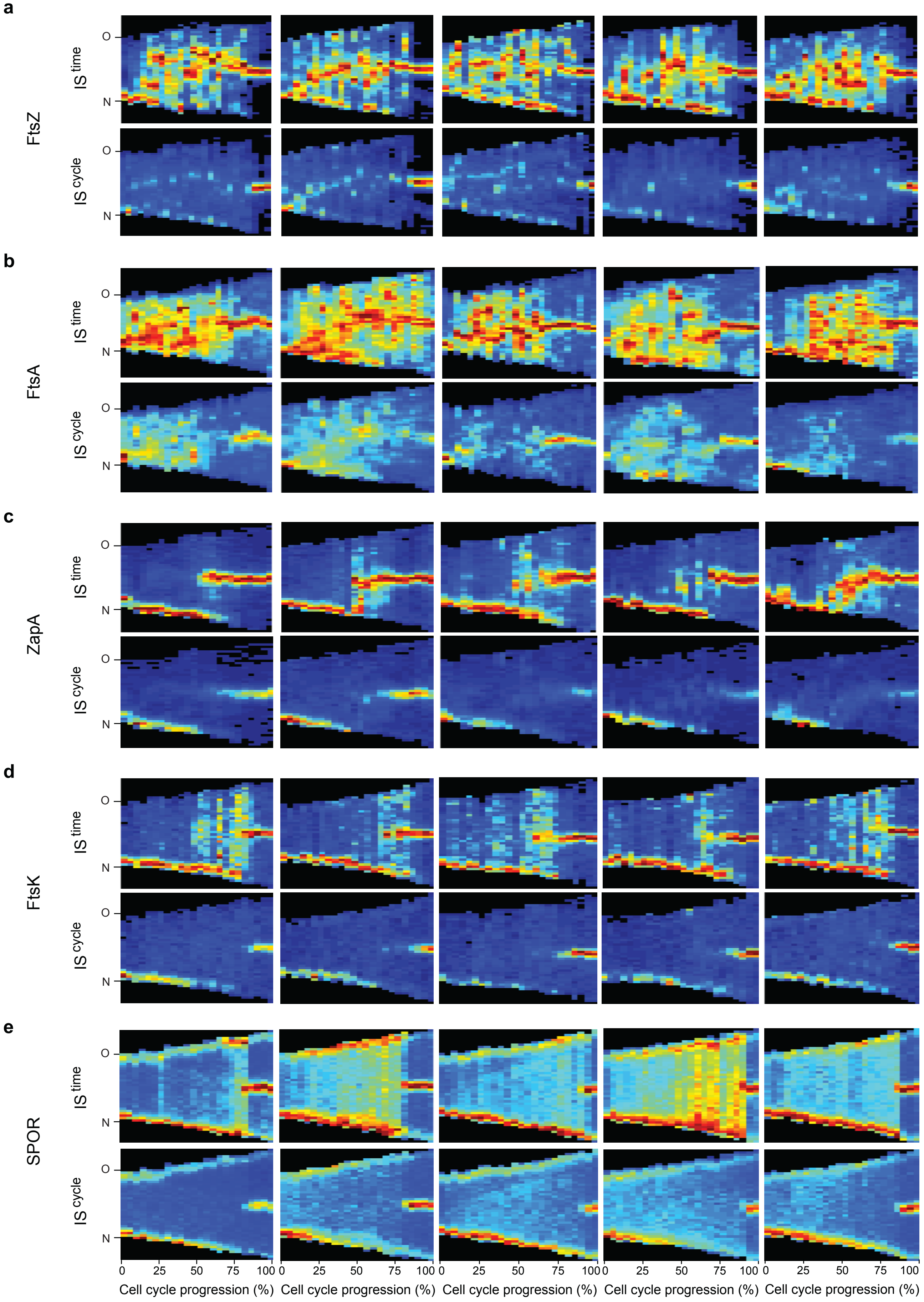


Supplementary Figure 4

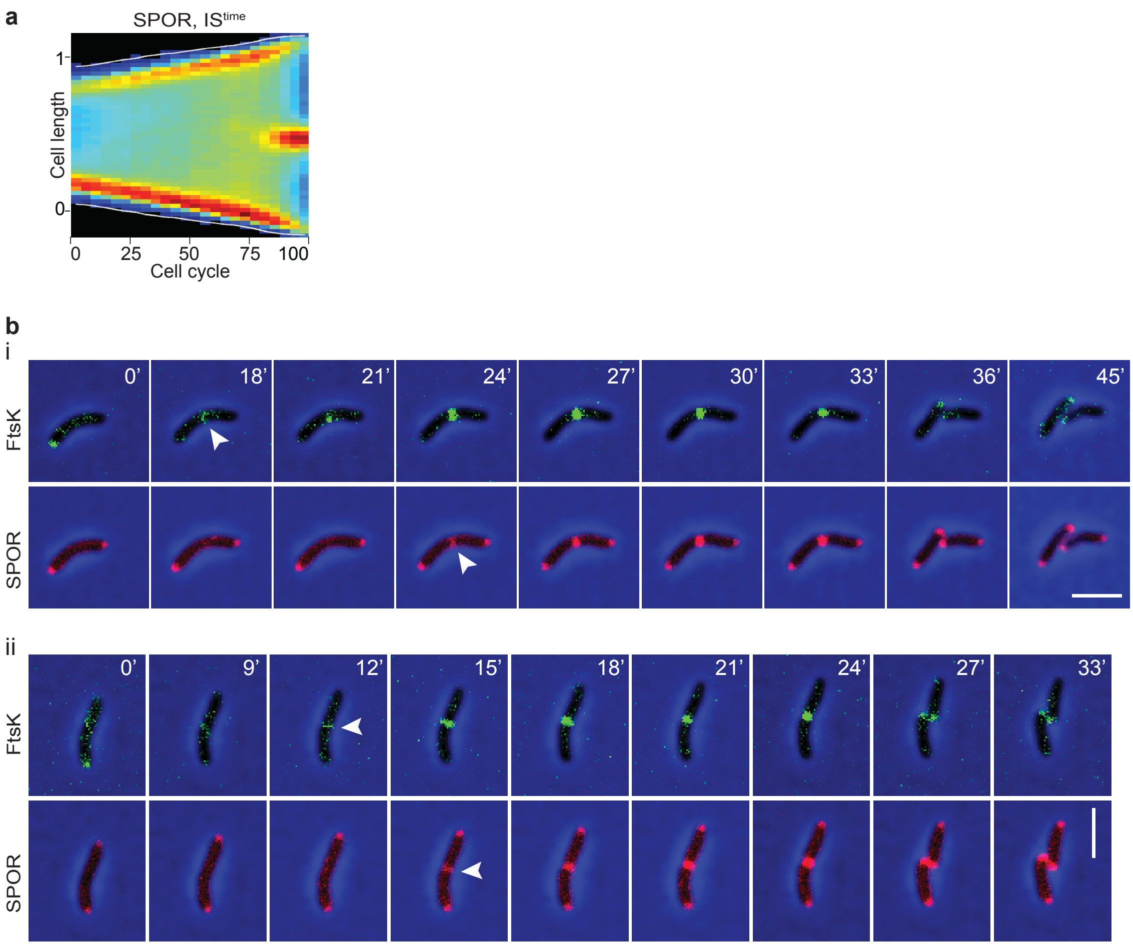


Supplementary Figure 5

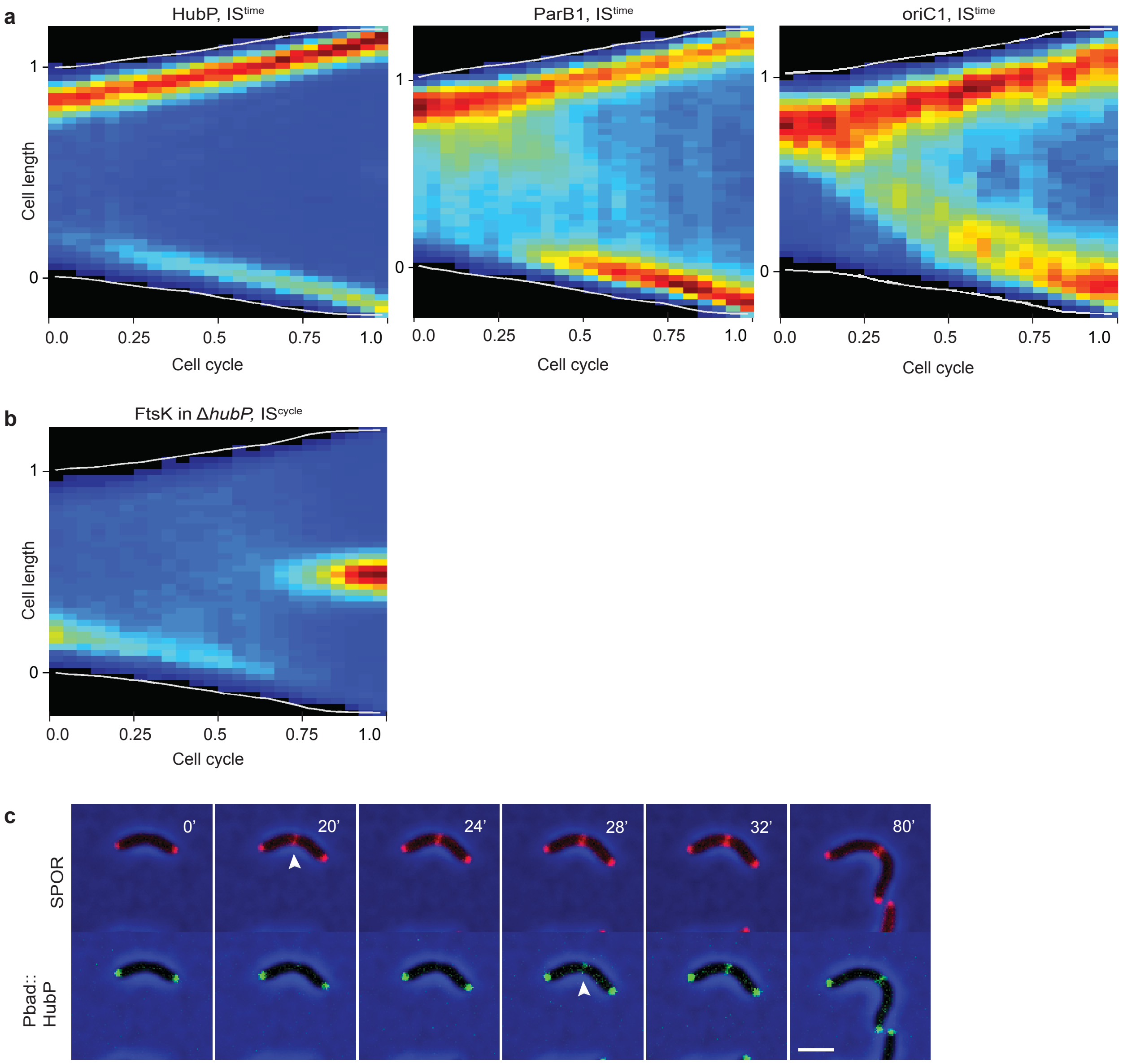


Supplementary Figure 6

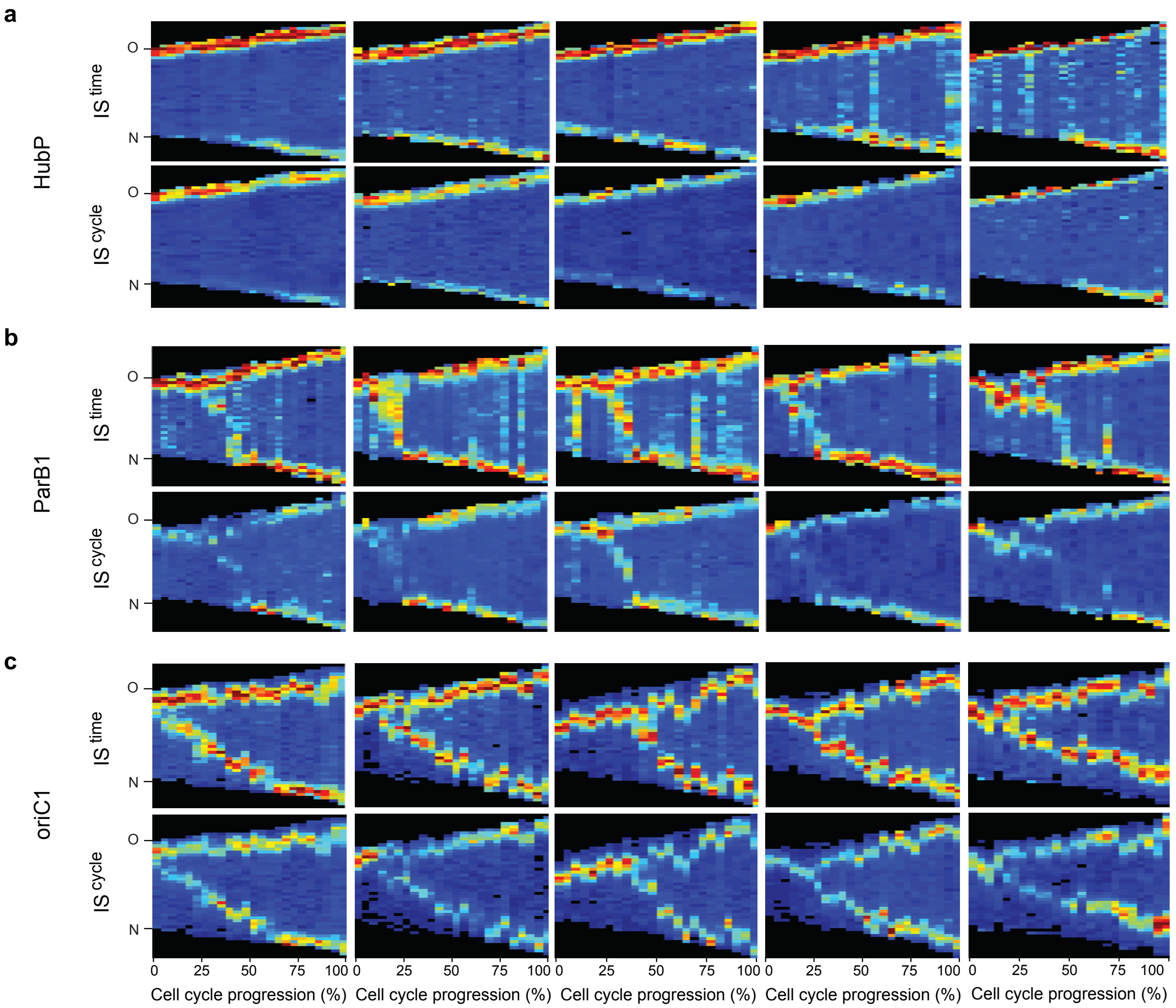


Supplementary Figure 7

**Supplementary Table 1**. List of bacterial strains and plasmids

| **Strains** | | |
|---|---|---|
| **Name** | **Relevant genotype or features** | **Reference** |
| EGV10 | N16961 *ChapR* Δ*lacZ::*($P_{BAD}$*::ftsZ-RFPT-Sh ble*) zeo$^R$ gm$^R$ | (1) |
| EGV11 | N16961 *ChapR* Δ*lacZ::*($P_{BAD}$*::RFPT-zapA-Sh ble*) zeo$^R$ gm$^R$ | This study |
| EGV27 | N16961 Δ*lacZ::*($P_{BAD}$*::ftsZ-RFPT-Sh ble*) Δ*hapR::*($P_{lac}$*::lacI-YGFP-cat*) + *lacO* array-*aph* inserted on chr1 at position 0.05 Mb (53355 bp) (oriC1) zeo$^R$ gm$^R$ cml$^R$ kan$^R$ | This study |
| EGV34 | N16961 *ChapR* Δ*lacZ* $P_{ftsK}$*::ftsK-YGFP-Sh ble* zeo$^R$ gm$^R$ | (1) |
| EGV37 | N16961 Δ*lacZ* $P_{ftsK}$*::ftsK-YGFP-Sh ble* Δ*hapR::*($P_{BAD}$*::ftsZ-RFPT-cat*) zeo$^R$ gm$^R$ cml$^R$ | (1) |
| EGV54 | N16961 *ChapR* Δ*lacZ minCD::Sh ble* zeo$^R$ gm$^R$ | (1) |
| EGV64 | N16961 Δ*lacZ::*($P_{BAD}$*::YGFP-ftsA-Sh ble*) zeo$^R$ gm$^R$ | This study |
| EGV72 | N16961 *ChapR* Δ*lacZ::*($P_{lac}$*::YGFP-parB1-Sh ble*) zeo$^R$ gm$^R$ | This study |
| EGV77 | N16961 *ChapR* Δ*lacZ::*($P_{BAD}$*::YGFP-ftsL-Sh ble*) zeo$^R$ gm$^R$ | This study |
| EGV100 | N16961 *ChapR* Δ*lacZ* Δ*hubP minCD::Sh ble* zeo$^R$ gm$^R$ | (1) |
| EGV155 | N16961 *ChapR* Δ*lacZ::*($P_{BAD}$*::dsbA$_{ss}$-mCherry-SPOR-Sh ble*) SPOR domain of *E. coli* FtsN (FtsN[243-319]) (signal sequence from DsbA) zeo$^R$ gm$^R$ | This study |
| EGV157 | N16961 *ChapR* Δ*lacZ::*($P_{BAD}$*::hubP-YFP-Sh ble*) zeo$^R$ gm$^R$ | This study |
| EGV357 | N16961 *ChapR* Δ*lacZ::*($P_{BAD}$*::RFPT-zapA-Sh ble*) carrying the *ftsZ ts* chromosomal mutation (G106S) zeo$^R$ gm$^R$ | This study |
| EGV417 | N16961 *ChapR* Δ*lacZ* $P_{ftsK}$*::ftsK-YGFP-Sh ble* carrying the *ftsZ ts* chromosomal mutation (G106S) zeo$^R$ rif$^R$ gm$^R$ | This study |
| EGV418 | N16961 *ChapR* Δ*lacZ::*($P_{BAD}$*::YGFP-ftsA-Sh ble*) carrying the *ftsZ ts* chromosomal mutation (G106S) zeo$^R$ gm$^R$ | This study |
| EGV419 | N16961 *ChapR* Δ*lacZ::*($P_{BAD}$*::YGFP-ftsL-Sh ble*) carrying the *ftsZ ts* chromosomal mutation (G106S) zeo$^R$ gm$^R$ | This study |
| EGV420 | N16961 *ChapR* Δ*lacZ::*($P_{BAD}$*::YGFP-ftsI-Sh ble*) carrying the *ftsZ ts* chromosomal mutation (G106S) zeo$^R$ gm$^R$ | This study |
| EGV422 | N16961 *ChapR* Δ*lacZ::*($P_{BAD}$*::dsbA$_{ss}$-mCherry-SPOR-Sh ble*) carrying the *ftsZ ts* chromosomal mutation (G106S) SPOR domain of *E. coli* FtsN (FtsN[243-319]) (signal sequence from DsbA) zeo$^R$ gm$^R$ | This study |
| EGV429 | N16961 Δ*lacZ* $P_{ftsK}$*::ftsK-YGFP-Sh ble* Δ*hapR::*($P_{BAD}$*::dsbA$_{ss}$-mCherry-SPOR-cat*) SPOR domain of *E. coli* FtsN (FtsN[243-319]) (signal sequence from DsbA) zeo$^R$ gm$^R$ cml$^R$ | This study |
| EGV430 | N16961 Δ*lacZ::*($P_{BAD}$*::hubP-YFP-Sh ble*) Δ*hapR::*($P_{BAD}$*::dsbA$_{ss}$-mCherry-SPOR-cat*) SPOR domain of *E. coli* FtsN (FtsN[243-319]) (signal sequence from DsbA) zeo$^R$ gm$^R$ cml$^R$ | This study |
| EGV437 | N16961 *ChapR* Δ*lacZ* $P_{ftsK}$*::1ftsK-YGFP-Sh ble* $P_{hubP}$*::hubP-RFPT-aadA* zeo$^R$ spec$^R$ gm$^R$ | This study |
| EGV438 | N16961 *ChapR* Δ*lacZ* $P_{hubP}$*::hubP-RFPT-aadA* carrying the *ftsZ ts* chromosomal mutation (G106S) spec$^R$ rif$^R$ gm$^R$ | This study |
| EGV439 | N16961 *ChapR* Δ*lacZ::*($P_{lac}$*::sfGFP-ftsN-Sh ble*) carrying the *ftsZ ts* chromosomal mutation (G106S) zeo$^R$ gm$^R$ | This study |
| EGV441 | N16961 *ChapR* Δ*lacZ* Δ*hubP* $P_{ftsK}$*::ftsK-YGFP-Sh ble* zeo$^R$ gm$^R$ | This study |
| EPV50 | N16961 *ChapR* Δ*lacZ* gm$^R$ | (2) |
| EPV390 | N16961 *ChapR* Δ*lacZ* carrying the *ftsZ ts* chromosomal mutation (G106S) rif$^R$ gm$^R$ | (1) |
| EPV430 | N16961 *ChapR* Δ*lacZ::*($P_{lac}$*::sfGFP-ftsN-Sh ble*) zeo$^R$ gm$^R$ | This study |
| EPV432 | N16961 *ChapR* Δ*lacZ* carrying the *ftsI ts* chromosomal mutation (Y388D) rif$^R$ gm$^R$ | This study |
| EPV437 | N16961 *ChapR* Δ*lacZ::*($P_{BAD}$*::ftsZ-RFPT-Sh ble*) carrying the *ftsI ts* chromosomal mutation (Y388D) zeo$^R$ gm$^R$ | This study |
| EPV439 | N16961 *ChapR* Δ*lacZ::*($P_{BAD}$*::dsbA$_{ss}$-mCherry-SPOR-Sh ble*) carrying the *ftsI ts* chromosomal mutation (Y388D) SPOR domain | This study |

| | | |
|---|---|---|
| | of *E. coli* FtsN (FtsN[243-319]) (signal sequence from DsbA) zeo$^R$ gm$^R$ | |
| EPV443 | N16961 *ChapR* Δ*lacZ* P$_{hubP}$*::hubP-RFPT-aadA* carrying the *ftsI ts* chromosomal mutation (Y388D) spec$^R$ rif$^R$ gm$^R$ | This study |
| EPV447 | N16961 *ChapR* Δ*lacZ::*(P$_{lac}$*::sfGFP-ftsI-Sh ble*) zeo$^R$ gm$^R$ | This study |
| EPV449 | N16961 *ChapR* Δ*lacZ::*(P$_{lac}$*::sfGFP-ftsI-Sh ble*) carrying the FtsI *ts* chromosomal mutation (Y388D) zeo$^R$ gm$^R$ | This study |
| EPV453 | N16961 *ChapR* Δ*lacZ* P$_{hubP}$*::hubP-sfGFP* gm$^R$ | This study |
| EPV454 | N16961 *ChapR* P$_{hubP}$*::hubP-sfGFP* Δ*lacZ::*(P$_{BAD}$*::dsbA*$_{ss}$*-mCherry-SPOR-Sh ble*) SPOR domain of *E. coli* FtsN (FtsN[243-319]) (signal sequence from DsbA) zeo$^R$ gm$^R$ | This study |
| EPV455 | N16961 *ChapR* Δ*lacZ* P$_{hubP}$*::hubP-sfGFP minCD::Sh ble* zeo$^R$ gm$^R$ | This study |
| | | |
| **Plasmids** | | |
| **Name** | **Relevant genotype or features** | **Reference** |
| pAD20 | integration-excision vector; Tet'-*lacO* array-*aph* -'Tet; *sacB*; ori R6K; cml$^R$, kan$^R$ | (2) |
| pEG233 | P$_{BAD}$*::ftsZ-RFPT-Sh ble* flanked by the upstream and downstream regions of *lacZ*; ori pUC; amp$^R$, zeo$^R$ | (1) |
| pEG234 | P$_{BAD}$*::RFPT-zapA-Sh ble* flanked by the upstream and downstream regions of *lacZ*; ori pUC; amp$^R$, zeo$^R$ | (1) |
| pEG242 | P$_{BAD}$*::YGFP-ftsI-Sh ble* flanked by the upstream and downstream regions of *lacZ*; ori pUC; amp$^R$, zeo$^R$ | This study |
| pEG245 | P$_{lac}$*::lacI-YGFP-cat* flanked by the upstream and downstream regions of *hapR*; ori pUC; amp$^R$, cml$^R$ | (1) |
| pEG248 | *ftsK-YGFP-Sh ble* flanked by the upstream and downstream regions of *ftsK*; ori pSC101; amp$^R$, zeo$^R$ | (1) |
| pEG252 | *Sh ble* flanked by the upstream and downstream regions of *minCD*; ori pUC; amp$^R$, zeo$^R$ | (1) |
| pEG266 | *Sh ble* flanked by the upstream and downstream regions of *hubP*; ori pUC; amp$^R$, zeo$^R$ | This study |
| pEG270 | P$_{BAD}$*::YGFP-ftsA-Sh ble* flanked by the upstream and downstream regions of *lacZ*; ori pUC; amp$^R$, zeo$^R$ | This study |
| pEG282 | P$_{lac}$*::YGFP-parB1-Sh ble* flanked by the upstream and downstream regions of *lacZ*; ori pUC; amp$^R$, zeo$^R$ | This study |
| pEG287 | P$_{BAD}$*::YGFP-ftsL-Sh ble* flanked by the upstream and downstream regions of *lacZ*; ori pUC; amp$^R$, zeo$^R$ | This study |
| pEG323 | P$_{BAD}$*::dsbA*$_{ss}$*-mCherry-SPOR-Sh ble* flanked by the upstream and downstream regions of *lacZ*; SPOR domain of *E. coli* FtsN (FtsN[243-319]) (signal sequence from DsbA); ori pUC; amp$^R$, zeo$^R$ | This study |
| pEG324 | P$_{BAD}$*::hubP-YFP-Sh ble* flanked by the upstream and downstream regions of *lacZ*; ori pUC; amp$^R$, zeo$^R$ | This study |
| pEG392 | P$_{BAD}$*::dsbA*$_{ss}$*-mCherry-SPOR-cat* flanked by the upstream and downstream regions of *hapR*; SPOR domain of *E. coli* FtsN (FtsN[243-319]) (signal sequence from DsbA); ori pUC; amp$^R$, cml$^R$ | This study |
| pEG396 | P$_{lac}$*::sfGFP-ftsN-Sh ble* flanked by the upstream and downstream regions of *lacZ*; ori pUC; amp$^R$, zeo$^R$ | This study |
| pEG398 | P$_{lac}$*::sfGFP-ftsI-Sh ble* flanked by the upstream and downstream regions of *lacZ*; ori pUC; amp$^R$, zeo$^R$ | This study |
| pEG400 | integration-excision vector; *hubP-RFPT-aadA* at native locus; *sacB*; ori R6K; amp$^R$, spec$^R$ | This study |
| pEYY24 | integration-excision vector; *hubP-sfGFP* at native locus; *sacB*; ori R6K; amp$^R$ | Y. Yamaichi gift |

## References


1. **Galli E**, **Poidevin M**, **Le Bars R**, **Desfontaines J-M**, **Muresan L**, **Paly E**, **Yamaichi Y**, **Barre F-X**. 2016. Cell division licensing in the multi-chromosomal Vibrio cholerae bacterium. Nat Microbiol **1**:16094.

2. **David A**, **Demarre G**, **Muresan L**, **Paly E**, **Barre F-X**, **Possoz C**. 2014. The two Cis-acting sites, parS1 and oriC1, contribute to the longitudinal organisation of Vibrio cholerae chromosome I. PLoS Genet **10**:e1004448.
